# Molecular regulation of tomato male reproductive development

**DOI:** 10.1007/s42994-022-00094-1

**Published:** 2023-02-04

**Authors:** Dandan Yang, Zhao Wang, Xiaozhen Huang, Cao Xu

**Affiliations:** 1 State Key Laboratory of Plant Genomics, National Center for Plant Gene Research (Beijing), Institute of Genetics and Developmental Biology, The Innovative Academy of Seed Design, Chinese Academy of Sciences, Beijing, 100101 China; 2 CAS-JIC Centre of Excellence for Plant and Microbial Science, Institute of Genetics and Developmental Biology, Chinese Academy of Sciences, Beijing, 100101 China; 3 University of Chinese Academy of Sciences, Beijing, 100049 China

**Keywords:** Tomato, Climate change, Male, Reproductive development

## Abstract

The reproductive success of flowering plants, which directly affects crop yield, is sensitive to environmental changes. A thorough understanding of how crop reproductive development adapts to climate changes is vital for ensuring global food security. In addition to being a high-value vegetable crop, tomato is also a model plant used for research on plant reproductive development. Tomato crops are cultivated under highly diverse climatic conditions worldwide. Targeted crosses of hybrid varieties have resulted in increased yields and abiotic stress resistance; however, tomato reproduction, especially male reproductive development, is sensitive to temperature fluctuations, which can lead to aborted male gametophytes, with detrimental effects on fruit set. We herein review the cytological features as well as genetic and molecular pathways influencing tomato male reproductive organ development and responses to abiotic stress. We also compare the shared features among the associated regulatory mechanisms of tomato and other plants. Collectively, this review highlights the opportunities and challenges related to characterizing and exploiting genic male sterility in tomato hybrid breeding programs.

## Introduction

Flowering plants are among the most successful living organisms at least partly because of their morphological diversity and ability to grow in various ecological niches, which is primarily because of reproductive innovations (Chen et al. 2019; Ge et al. 2010). Reproductive development is crucial for the maintenance of genetic diversity and involves complex processes during diploid and haploid phases, including male and female organogenesis, meiosis, gametogenesis, pollination, and fertilization (Ke et al. 2021; Liu et al. 2021; Ma 2005). Fruits and seeds are two major components of human diets; their production depends on reproductive development-related activities (Gao et al. 2015; Li et al. 2018b). During plant reproduction, the male reproductive organ (i.e., anther and filament) undergoes specific changes, including anther differentiation, functional pollen production, and anther dehiscence, ultimately resulting in the release of mature pollen. The failure of any of these processes may lead to male sterility, limited reproduction, and decreased crop production. Moreover, as sessile organisms, plants are highly susceptible to environmental factors, with the reproductive stage (especially male reproduction-related processes) more sensitive to abiotic stress than the vegetative growth stage (Zhang et al. 2021). An exposure to abiotic stress may impair anther and pollen development, resulting in male sterility and low crop yields. Clarifying plant responses to abiotic stress during the male gametophytic phase is critical for enhancing crop productivity. Additionally, male-sterile varieties are valuable resources, because they may be used to produce hybrids. Modulating male reproductive development may facilitate the efficient use of biotechnology-based male sterility for the selective breeding and commercial development of hybrid lines (Chen and Liu 2014).

Tomato, which is one of the most important vegetable crops, is cultivated worldwide. Although tomato plants can grow under various climatic conditions, their reproductive development, especially male reproductive development, is severely impeded by abiotic stresses, resulting in decreased yields and relatively low fruit quality (Gerszberg and Hnatuszko-Konka 2017). Many studies conducted over the last few decades to maintain or increase tomato production focused on anther and pollen development. We herein review the cytological and morphological changes associated with tomato male organ development, the molecular and genetic pathways influencing tomato male reproduction-related activities, and the general mechanisms by which abiotic stresses can inhibit tomato male reproductive development. We also describe experimental strategies useful for enhancing tomato male reproductive development under abiotic stress conditions.

## Tomato male reproductive development

**Fig. 1:**
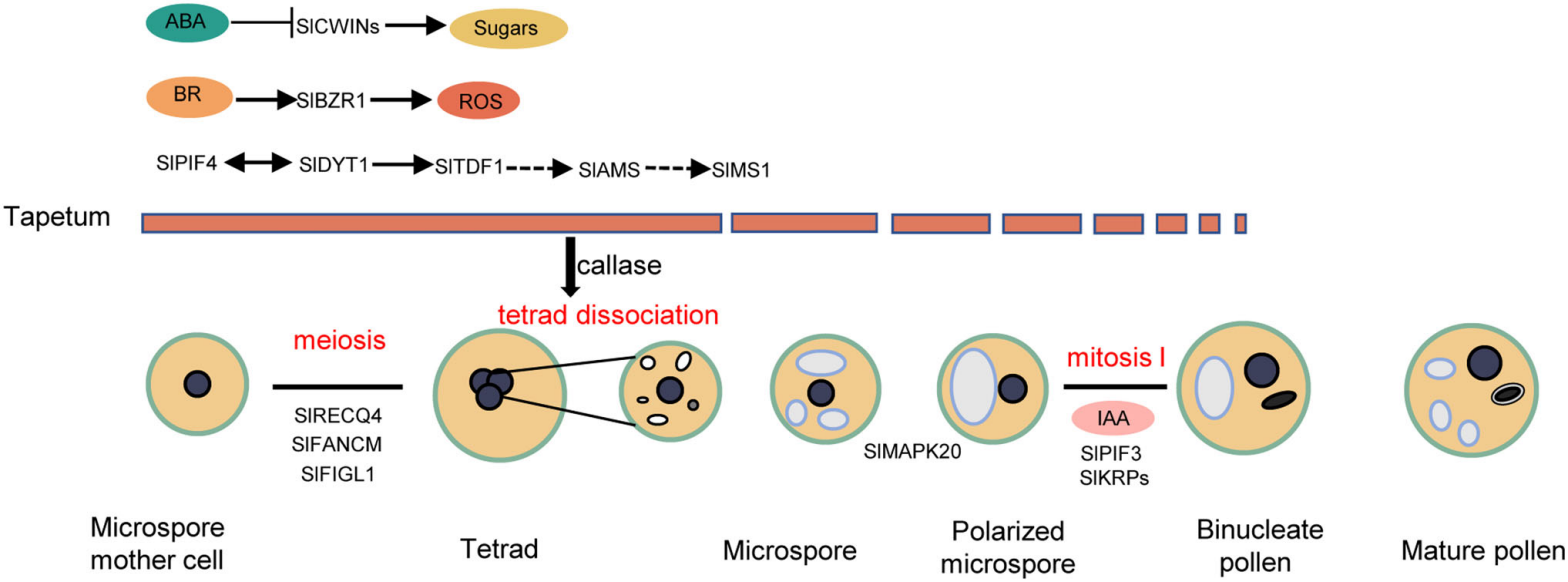
Schematic overview of tomato anther and pollen development. SlPIF4-SlDYT1-SlTDF1-SlAMS-SlMS1, ROS, and sugar pathways affect tomato tapetum development. SlRECQ4, SlFANCM, and SlGIGL1 participate in tomato meiosis. SlPIF3, SlKRPs, and IAA regulate pollen mitosis I. The dashed line indicates a putative role or relationship

After producing 8–10 leaves, the shoot apical vegetative meristem of tomato plants transforms into an inflorescence meristem, which ultimately forms a lateral monochasial inflorescence that includes 6–10 flowers (Huang et al. 2018; Park et al. 2012). Tomato flowers typically consist of five sepals and five petals that are arranged in an alternating pattern. The five anti-sepalous stamens are fused together to form a cone around the style inside of the petals. On the basis of morphological and cytological characteristics, the tomato floral developmental process has been divided into 20 stages (Brukhin et al. 2003). The stamen primordia are detectable at stage 3 after the sepal and petal primordia have been initiated, but before the initiation of carpel primordia, and initially form as a whorl of small bumps at a specific site on the surface of the floral meristem (Brukhin et al. 2003). During these processes, the division of the floral meristem L1, L2, and L3 layers produces specialized stamen cells and tissues (Goldberg et al. 1993). More specifically, the L1 layer cells develop into the epidermis and stomium, whereas the L3 layer cells produce the connective, vascular bundle, and circular cell clusters adjacent to the stomium. Meanwhile, the periclinal division of the L2 layer cells results in the initiation of the anther primordia, which subsequently differentiate into the archesporial cells and generate the inner microspore mother cells and the outer parietal cell layer (outer to inner layers: endothecium, middle layer, and tapetum). The tapetum is a single layer of metabolically active cells and most obvious anther cell layer. Of the two basic tapetum types, the amoeboid tapetum extends into the locule encasing the microspore to provide the microspore with required materials (e.g., in *Arum* species or *Cichorium intybus*), whereas the secretory tapetum, which is more common among plants (e.g., *Arabidopsis thaliana*, rice, and tomato), provides nutrients through the liquid in the locule that bathes the developing microspore (Pacini 2010). The tapetum is a nutritive somatic tissue accompanying with the pollen development by providing nutrition to microspores, enzymes for microspores release, precursors for pollen wall formation and small RNAs to regulate germline cells (Ma et al. 2021; Santiago et al*.* 2019; Shi et al. 2015; Wang et al. 2018; Yao et al. 2022). Programmed cell death (PCD)-triggered tapetal cells degradation plays a vital role in nutrition supply, which often occurs synchronously with post-meiotic microspore development and is tightly controlled by integration of internal and external signals (Parish et al. 2012). In tomato, tapetum degradation is initiated before the tetrad stage and is completed at the bicellular pollen stage (Fig. 1). Recent research on tomato revealed that premature or delayed tapetum degeneration usually results in male sterility (Pan et al. 2021; Yan et al. 2020; Yang et al. 2021).

In addition to the outer layer of parietal cells, the inner microspore mother cells also contribute to pollen development, which is one of the most basic biological processes related to plant sexual reproduction. Pollen development involves the following two major stages: microsporogenesis (i.e., differentiation of sporogenous cells and meiosis) and microgametogenesis (i.e., post-meiotic microspore development) (Gómez et al. 2015). In tomato, microspore mother cells have a relatively large nucleus and are surrounded by parietal cells at floral developmental stage 7 (Fig. 1). The sporogenous cells undergo meiosis at stage 9 and produce a callose-encased tetrad. At stage 10, the callose is hydrolyzed by β-1, 3-glucanase secreted from tapetal cells, resulting in the release of free microspores from the tetrad (Fig. 1) (Brukhin et al. 2003; Pan et al. 2021). The released microspores continue to develop using nutrients from the degenerated tapetal cells. The formation of vacuoles within microspores is accompanied by the migration of the nucleus to one side of the cell (Fig. 1). Vacuolated microspores undergo an asymmetrical mitotic division to generate pollen grains that comprise two cells with differing characteristics. The larger vegetative cell has a dispersed nucleus and more cytoplasm, whereas the smaller generative cell, which is enclosed entirely by the vegetative cell, contains condensed chromatin and relatively little cytoplasm (Fig. 1). As pollen grains mature, a break in the stomium that leads to pollen release is due to the degeneration of the tapetum and the reinforcement of the exothecium. In tomato, the generative cell of the released pollen grains divides into two sperm cells during pollen mitosis II (PMII) in the pollen tube that grows through the pistil. In contrast, in *Arabidopsis* and rice, PMII occurs in the anther sacs (Borg et al. 2009; McCormick 2004).

## Genes involved in tomato male reproductive development

Male reproductive development involves multiple stages, with abnormalities at any stage potentially leading to male sterility (i.e., structural, functional, or sporogenous male-sterile mutants) (Kaul 2012). In tomato, structural male-sterile mutants usually have extremely deformed stamens unable to produce pollen. In contrast, functional male-sterile mutants form viable pollen grains that cannot reach the stigma because of a protruding style or indehiscent anthers. *Stamenless* (*sl*) was the first verified structural male-sterile tomato mutant, with vestigial stamens adhering to the carpels (Bishop 1954). The tomato *spl-like* mutant is indistinguishable from wild-type tomato in terms of vegetative development, but its anthers have a filamentous structure and do not produce pollen, leading to male sterility (Rojas-Gracia et al. 2017). In *Arabidopsis*, the transcription factors *sporocyteless*/*nozzle* (*spl*/*nzz*) mutants were initially identified as sterile mutants, which displayed failure of male and female gametophyte formation (Schiefthaler et al. 1999; Yang et al. 1999). Additionally, SPL/NZZ had also been demonstrated as a transcriptional repressor during *Arabidopsis* ovule development as the C-terminal end of SPL/NZZ contains a typical EAR motif (ERF-associated amphiphilic repression), a well-characterized repression domain (Ohta et al. 2001; Wei et al. 2015). A MADS-box transcription factor AGAMOUS (AG) that defines stamens and carpels regulates microsporogenesis and pollen formation by activating the expression of SPL/NZZ (Ito et al. 2004; Schiefthaler et al. 1999; Yang et al. 1999). Earlier research confirmed that SlDEFICIENS (SlDEF) is necessary for the normal development of petal and stamen characteristics, while tomato MADS-box 6 (SlTM6) modulates stamen morphology (Cao et al. 2019; De Martino et al. 2006). In tomato, stigma exsertion has been observed in functional male-sterile mutants (i.e., positional sterility). A recent investigation indicated that stigma exsertion is due to different genes in diverse tomato genotypes, including genes at several loci associated with long styles (e.g., *se2.1*, *StyleD1*, and *sty 8.1*) (Cheng et al. 2021). A mutation to a polygalacturonase-encoding gene, *ps-2*, results in non-dehiscent anthers in tomato (Gorguet et al. 2009). Some mutants with abnormal jasmonic acid and ethylene metabolism exhibit impaired tomato anther dehiscence (Schubert et al. 2019).

Most tomato male-sterile mutants exhibit sporogenous male sterility. Accordingly, they have morphologically normal flowers that produce little or no viable pollen (Gorman et al. 1997). The tomato male-sterile mutant *ms10*^*35*^ resulting from a spontaneous mutation shows degenerating microspores with enlarged vacuoles during meiosis. A gene mapping analysis revealed that *Ms10*^*35*^ encodes a bHLH transcription factor and is homologous to the *Arabidopsis* gene *DYSFUNCTIONAL TAPETUM 1* (*DYT1*) (Jeong et al. 2014). This gene encodes a transcription factor that functions downstream of SPL/NZZ, but is one of the earliest tapetal development-related genes to be activated after another cells are initiated (Zhang et al. 2006). Additionally, DYT1 regulates the expression of many tapetal genes, such as *TAPETAL DEVELOPMENT1* (*TDF1*) and *ABORTED MICROSPORES* (*AMS*), the latter of which encodes a master regulator of tapetal development involved in the synthesis of lipidic and phenolic components essential for pollen wall patterning and flavonoid production (Gu et al. 2014; Sorensen et al. 2003; Zhu et al. 2008). In the *ms10*^*35*^ mutant, the expression levels of Solyc03g113530, Solyc08g062780, and Solyc04g008420, which are homologs of *AtTDF1*, *AtAMS*, and *MALE STERILITY1* (*AtMS1*), respectively, are downregulated. In addition to *ms10*^*35*^, the tomato *male sterility 32* (*ms32*) mutant fails to undergo meiosis; its mutated locus was mapped to a putative gene (Solyc01g081100) homologous to the *Arabidopsis bHLH89/91* gene (Liu et al. 2019). Moreover, the Solyc01g081100 expression level is downregulated in *ms10*^*35*^, suggesting that bHLH89/91 function downstream of DYT1 in tomato. In *Arabidopsis*, AMS has been reported to interact with bHLH89/91 to regulate the expression of *MYB80*, which promotes the sporopollenin synthesis for pollen wall formation (Ferguson et al. 2017; Lou et al. 2014; Wang et al. 2018; Xiong et al. 2016; Zhang et al. 2007). The rice bHLH protein UNDEVELOPED TAPETUM (OsUDT1), a putative homolog of AtDYT1, acts after initiation of the tapetum in an analogous manner to AtDYT1 (Jung et al. 2005). Moreover, a couple of important genes that are essential for tapetum development have been cloned from male-sterile rice mutants, including *TAPETUM DEGENERATION RETADATION* (*TDR*) and *OsMYB80*, which are homologs of *AtAMS* and *AtMYB80*, respectively (Han et al. 2021; Pan et al. 2020; Phan et al. 2011; Wilson and Zhang 2009). The functional similarity shared among these genes in both dicots and monocots suggest that the DYT1-TDF1-AMS-MYB80 transcriptional cascade might also play an essential and conserved roles in regulating tapetal development in tomato.

As mentioned above, during the establishment and specification of the anther cell layers, sporogenous cells encased in the tapetum are generated from archesporial cells. The sporogenous cells then undergo a conserved cell division necessary for eukaryotic sexual reproduction (i.e., meiosis), which leads to the generation of haploid microspores. Meiosis comprises the following five key stages: meiotic entry, recombination initiation, chromosome synapsis, resolution of recombination intermediates, and the second meiotic division (Ma 2005). Defective meiosis generally prevents the production of viable pollen grains. Genes involved in meiosis were identified in *Arabidopsis* and other crops following analyses of mutants that are sterile or less fertile than normal (Wang et al. 2021). Both Topoisomerase3α (TOP3α) and RecQ-mediated instability 1 (RMI1), which are components of the RTR (RecQ/Top3/Rmi1) complex, are crucial for meiosis; mutations to the corresponding *Arabidopsis* genes result in meiotic defects and sterility. Analyses of tomato plants indicated the mutation in the *top3a* mutant is lethal to embryos, whereas the mutation in the *rmi1* mutant does not cause abnormalities in somatic DNA repair or meiosis (Xing et al. 2012). Meiotic crossovers generate genetic diversity but improper crossover frequency can disrupt meiosis and cause pollen sterile in many plant species. A group of genes that exert the function for limiting meiotic recombination have been identified in *Arabidopsis,* including *Fanconi anemia of complementation group M* (*FANCM*), *Recombinant Escherichia coli ATP-dependent DNA helicase* (*RECQ*) and *AAAATPase FIDGETIN-LIKE-1* (*F**I**G**L1*). In contrast to the fact that loss of function of these genes individually increases the crossover frequency and has little effect on fertility in *Arabidopsis* (Mieulet et al. 2018), the rice *Osfigl1* mutant gives rise to aborted pollen, which may result from abnormal chromosome behavior during meiosis (Zhang et al. 2017). Similarly, the tomato *Slfigl1* mutant is also completely sterile, supporting the essential role of SlFIGL1 for fertility (Mieulet et al. 2018). To date, a few studies have been conducted on meiosis in tomato, which has resulted in the identification and characterization of several genes involved in meiosis. For example, silencing *MUTS-HOMOLOG 2* (*MSH2*), which encodes a protein that recognizes and repairs errors in DNA sequences, disrupts tomato male meiosis where half of the meiocytes stalled at the zygotene stage or combined to form diploid tetrads, which substantially inhibits normal pollen formation (Sarma et al. 2018; Wang et al. 2021).

The post-meiosis pollen developmental stage is microgametogenesis, in which microspores form pollen grains via cellular mitosis. This process depends on the asymmetrical division of the microspore during pollen mitosis I (PMI), which is essential for establishing male germ cells (McCormick 2004). During mitosis in somatic cell, the division site is marked by a circumferential band of microtubules, called the preprophase band. Although no obvious preprophase bands are observed in microspores before division, the migration of microspore nuclear is sensitive to colchicine, suggesting the involvement of a microtubule system (Twell et al. 1998). Compelling evidence comes from studies of the orchid *Phalaenopsis*, in which a specialized generative pole microtubule system appears at the future generative cell pole prior to nuclear migration (Brown and Lemmon 1991). In *Arabidopsis*, mutations in microtubule-related genes impact asymmetrical division of microspores and cause abnormal male germline formation, ultimately lead to male sterility, such as *gemini pollen1* (*gem1*), *two-in-one* (*tio*) and *kinesin- 12A/ 12B* (Liu and Qu 2008; Twell 2011). Given that the microtubule system plays an important role in directing and maintaining nuclear migration, one can expect that it might act in response to cellular signals or polarity determinants within the cytoplasm. However, the underlying mechanisms are still unclear so far. Mutations to cell-cycle regulators in *Arabidopsis* can impair the progression of pollen mitosis with lethal consequences for gametophytes (Liu and Qu 2008; Liu et al. 2008; Takatsuka et al. 2015). Loss-of-function of genes related to microtubules or cell-cycle regulators in tomato also directly affects the asymmetrical division of pollen cells (Yang et al. 2022). In addition, some intracellular metabolites help regulate pollen mitosis. For example, in *Arabidopsis*, the auxin flow in anthers affects pollen development by regulating PMI (Feng et al. 2006). Additionally, the mutations in the *yuc2yuc6* double mutant result in arrested PMI and a lack of viable pollen (Yao et al. 2018). Thus, auxin is vital for asymmetrical pollen cell division. In tomato, a mutation to *SlPIF3* prevents the production of viable pollen grains because of the associated arrested PMI. Compared with wild-type tomato plants, the *Slpif3* mutant has a substantially lower anther auxin content and abnormal microtubule- and cell-cycle-related gene expression levels (Yang et al. 2022). The application of exogenous auxin downregulates the expression of cyclin kinase inhibitor genes (*SlKRP2* and *SlKRP4*), while also partially rescuing the pollen viability of the *Slpif3* mutant, suggesting that auxin regulates tomato PMI by modulating the expression of genes related to microtubules and the cell cycle. Furthermore, sugar metabolism-associated signaling pathways are involved in pollen mitosis in tomato. Recent research demonstrated that SlSWEET5b facilitates tomato pollen mitosis and maturation by mediating the transport of apoplasmic hexose into developing pollen cells (Ko et al. 2022), while SlMAPK20 is necessary for the uninucleate-to-binucleate transition of tomato pollen cells, because it regulates anther sugar metabolism (Chen et al. 2018). The molecular and regulatory mechanisms underlying the effects of sugar-related signaling pathways on pollen mitosis will need to be more precisely characterized in future studies.

Thus, the fine mapping of male sterility-related genes and the identification of genes associated with genic male sterility in tomato have deepened our understanding of the molecular basis of male reproductive development and may increase the utility of biotechnology-based male sterility systems in hybrid breeding programs.

## Adaptive responses of tomato male reproductive development to abiotic stresses

Extreme environmental stresses, including excessive heat, cold, and drought, adversely affect male fertility in flowering plants and cause substantial crop yield losses. For example, high-temperature stress can negatively affect male reproductive structures and processes (e.g., stigma exsertion or anther indehiscence), leading to decreased pollen dispersal and fruit set in tomato (Pan et al. 2019; Sato et al. 2002). In most plants, the processes from meiosis to PMI are especially vulnerable to abiotic stress. When pollen development reaches the meiotic stage, the tapetal cells are highly metabolically active, with 20- to 40-fold increases in mitochondrial activities (Parish et al. 2012). There is increasing evidence of the link between abiotic stress-induced male sterility and tapetal dysfunction (Gómez et al. 2015).

**Fig. 2:**
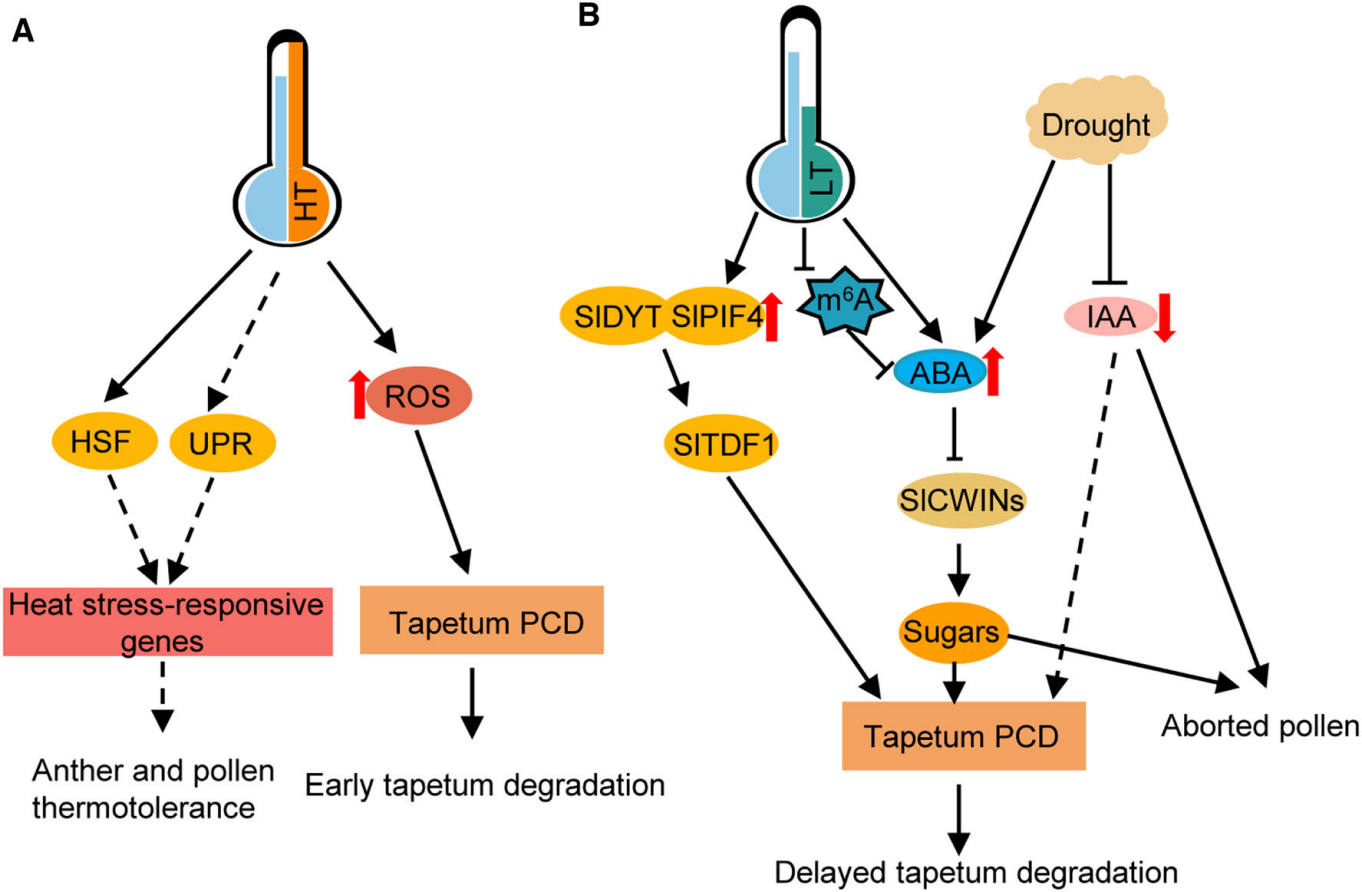
Regulatory pathway of the tomato anther and pollen development in response to abiotic stress. **A** High-temperature (HT) stress induces ROS production and the tomato tapetal PCD process, leading to male sterility. An exposure to HT stress also increases the *HSF* and *UPR* expression levels, thereby enhancing the ability of tomato anthers and pollen grains to tolerate heat stress. **B** Low-temperature (LT) stress leads to abnormal anther m^6^A levels and increased ABA contents in tomato, which adversely affects sugar metabolism by decreasing *CWIN* expression levels and delaying tapetal PCD degradation, ultimately resulting in male sterility. Moreover, LT stress enhances the production of SlPIF4, which interacts with SlDYT1 and induces *SlTDF1* expression, which leads to delayed tapetal PCD. Drought inhibits tapetal degradation through the ABA and IAA signaling pathways. Upward and downward pointing red arrows indicate significant increases and decreases, respectively. The dashed line indicates a putative role or relationship

Excessive heat is not the only temperature-related stress that can decrease crop yields. Even mild temperature fluctuations have induced grain yield losses of approximately 10%, 5.5%, and 3.8% per 1 °C increase in rice, wheat, and maize, respectively (Lobell et al. 2011; Peng et al. 2004; Tashiro and Wardlaw 1989). An exposure to heat stress can induce male sterility in *Arabidopsis*, rice, wheat, and tomato by impairing tapetum differentiation and microsporogenesis, activating tapetal PCD prematurely, and inhibiting the dehiscence of anthers (Parish et al. 2012). At the molecular level, heat stress induces oxidative damage, prevents proteins from folding correctly, and disturbs hormone homeostasis (Fig. 2A). In response to heat stress, mitochondria increase the production of reactive oxygen species (ROS) via enhanced aerobic metabolism and cause oxidative damage and cell death, with crucial effects on tapetal PCD in *Arabidopsis*, rice, and tomato (Parish et al. 2012; Zhang et al. 2021). A recent study showed that loss-of-function mutations to *DWARF* (*DWF*) and *BRASSINAZOLE-RESISTANT1* (*BZR1*) alter the timing of ROS production and delay tapetal PCD in tomato (Yan et al. 2020). These findings provide evidence that ROS homeostasis in anthers contributes to the regulation of tapetal cell degeneration. Heat stress promotes the production of protein disulfide isomerase 9 (PDI9), which affects nascent and misfolded proteins in the endoplasmic reticulum; a mutation to the corresponding gene decreases pollen viability at high temperatures (Feldeverd et al. 2020). Plants rely on diverse mechanisms to withstand heat stress, including the production of antioxidants and ROS scavengers as well as the induction of heat shock transcription factors (HSFs) and heat shock proteins (HSPs) (Chaturvedi et al. 2013). Heat stress may also activate a regulatory loop in which accumulating ROS modulate signaling cascades that produce antioxidant enzymes that eliminate excessive ROS in cells (Parish et al. 2012). Increases in ROS levels reportedly induce the production of detoxification-related enzymes in wheat pollen (Kumar et al. 2014). Additionally, increasing ROS contents can upregulate the expression of *HEAT SHOCK TRANSCRIPTION FACTOR A1* (*HsfA1*), which substantially activates the expression of heat responsive genes. In *Arabidopsis* and tomato *hsfa1* mutants, the expression levels of many HS-responsive genes are lower than normal, resulting in vegetative tissues with HS-insensitive phenotypes (Mishra et al. 2002; Yoshida et al. 2011). Many *HSF* and *HSP* genes are highly expressed in tomato microsporocytes and microspores, and their expression levels may be further increased by heat stress (Chaturvedi et al. 2013). For example, SlHsfA2 regulates the expression of several HS-responsive genes and maintains pollen viability in plants exposed to heat stress during meiosis in the microspore stage, suggestive of the importance of HsfA2 for pollen thermotolerance (Fragkostefanakis et al. 2016). The contribution of AtHsfA5 to pollen thermotolerance was revealed in an earlier study in which the pollen abortion rate was relatively high for the *Athsfa5* mutant because of defective HS responses (Reňák et al. 2014). Phytohormone signaling pathways typically mediate plant responses to abiotic stress. Excessive heat causes the abscisic acid (ABA) level to increase in rice anthers, whereas the IAA and GA contents decrease, leading to a decrease in pollen fertility (Zhang et al. 2021). The application of exogenous auxin can reverse the male sterility of barley plants exposed to high temperatures, demonstrating that auxin defection was an essential factor responsible for the heat stress-induced male sterility (Sakata et al. 2010). Notably, the AUXIN RESPONSE FACTOR17 (ARF17) was reported to directly regulate the expression of *CALLOSE SYNTHASE5* (*CalS5*), the key gene for callose biosynthesis. The miR160 regulated expression of *ARF17* is also required for its function during anthers development in *Arabidopsis* (Wang et al. 2017; Yang et al. 2013). Both ABA and GA are candidate signaling molecules that affect tapetal development by regulating carbohydrate availability in the tapetum and microspores under abiotic stresses (Zhang et al. 2021).

Cold stress is another prevalent abiotic factor affecting the growth and development of plants, especially subtropical vegetable crops. In *Arabidopsis* and rice, cold stress usually delays or inhibits tapetum regeneration by disrupting tapetal PCD, resulting in infertile pollen (Zhang et al. 2021). In developing tomato anthers and pollen grains, PCD-triggered tapetum degradation is initiated during the tetrad stage, intensifies from the early-to-late uninucleate stage, and is undetectable at the binucleate pollen stage. Cold stress in tomato leads to irregular hypertrophy and tapetum vacuolation because of delayed PCD, which subsequently leads to pollen abortion (Fig. 2B) (Pan et al. 2021; Yang et al. 2021). In contrast, pollen grains develop normally in the tomato *Slpif4* mutant under cold stress conditions because the inhibited activation of SlTDF1 makes the tapetum relatively insensitive to low temperatures (Fig. 2B) (Pan et al. 2021). The inhibition of invertase may be associated with tapetal hypertrophy and vacuolation. For example, during male gametogenesis in rice, low-temperature stress causes ABA to accumulate, which may suppress the expression of the tapetum-specific cell wall invertase gene *OsINV4* and the monosaccharide transporter genes *OsMST8* and *OsMST7*, leading to abnormalities in anther sugar metabolism and male sterility (Oliver et al. 2005). Abscisic acid has vital functions related to plant development and mediates responses to abiotic stress. Increased ABA levels improve plant abiotic stress tolerance during the vegetative growth stage, but there is a negative correlation between the anther ABA content and pollen fertility (Oliver et al. 2007). In tomato, an exposure to cold stress significantly increases anther ABA levels, but the expression of *SlCWIN7*, which is homologous to the rice gene *OsINV4*, significantly decreases, suggesting that low temperatures disrupt anther sugar metabolism, which leads to pollen sterility (Yang et al. 2021). Reversible epigenetic modifications typically occur in developing plants in response to environmental stress. At low temperatures, N6-methyladenosine (m^6^A) levels decrease in tomato anthers, which results in the altered transcription of many pollen development-related genes (Yang et al. 2021). These findings suggest m^6^A may influence tomato pollen development under cold conditions. Similar to cold stress, drought conditions can interfere with tapetal development by preventing or delaying the induction of PCD in developing tomato pollen grains (Lamin-Samu et al. 2021). Analyses of transcription levels and hormone metabolism showed that in tomato anthers, drought stress upregulates the expression of genes related to tapetum development and ABA homeostasis, whereas it has the opposite effect on the expression of sugar metabolism-associated genes, leading to increased ABA levels and decreased soluble sugar contents, which is consistent with what has been reported for other crops (Ji et al. 2011; Oliver et al. 2007). These results imply that in tomato, drought stress has detrimental effects on the metabolism of carbohydrates and hormones. The molecular mechanisms linking tapetal development and anther sugar and ABA homeostasis remain unclear. Future research will need to elucidate the cold- and/or drought-induced changes to these mechanisms that lead to tapetal dysfunction.

## Conclusions and future perspectives

Increases in the global population as well as climate changes are major issues that must be addressed to maintain agricultural production and food security. In addition to increasing crop yields, minimizing abiotic stress-induced production losses is a major objective among plant researchers. Thoroughly characterizing the mechanism regulating male fertility and identifying novel stress resistance genes associated with male reproductive development may enable the generation of stress-resistant germplasm resources suitable for biotechnology-based crop breeding. Research regarding reproductive stress tolerance has continued to progress because multiple strategies have been applied. For example, protein phase separations have been revealed to contribute to plant adaptive responses to cellular pH changes, temperature fluctuations, and oxidative stress. In *Arabidopsis*, a prion-like domain in ELF3 functions as a putative thermo-sensor to undergo protein phase transition that results in the formation of liquid droplets in response to increasing temperatures (Jung et al. 2020). The oxidation in the tomato shoot apical meristem triggers protein phase separations that enable TMF to bind to the promoter of the floral identity gene *ANANTHA* to repress its expression (Huang et al. 2021). The reversible protein phase separation promoted by changing internal and external conditions is responsible for the flexibility with which plants respond to global climate changes; this may represent a new abiotic stress mechanism influenced by plant developmental cues, especially those related to reproductive development. Stress resistance is typically a complex and polygenic trait. Developing novel plant lines with desirable traits through polygenic editing is a considerable challenge. There are numerous extant wild relatives of tomato that are highly tolerant to various stresses. Therefore, the de novo domestication of wild tomato species has been proposed as a viable alternative for creating climate-smart crops via genome engineering (Li et al. 2018a). Furthermore, a ‘two-in-one’ strategy-based breeding program that combines the production of male-sterile lines of an elite cultivar using CRISPR technology with the de novo domestication of wild species may enable researchers and breeders to enhance tomato stress resistance and yield (Xie et al. 2022). Although tomato male fertility has been widely studied and many recessive genic male-sterile mutants are useful for producing hybrid seeds, two major factors still restrict their commercial utility. First, it is difficult to efficiently maintain male sterility in genic male-sterile lines through self-pollination. Alternatively, analyses of male-sterile mutants in two lines may facilitate the large-scale isolation of pure male-sterile female lines via self-pollination (Chen and Liu 2014; Shi et al. 2021; Zhu et al. 2020). Second, the production of tomato hybrid seeds is an expensive and labor-intensive process. Because the stigma is completely covered by the staminal tube in tomato cultivars, hybridizations require the manual emasculation of the seed parent line. However, most tomato male-sterile mutants exhibit sporogenous male sterility and lack an exposed stigma, unlike functional male-sterile mutants. Elucidating tomato stamen morphological development and creating male-sterile lines with an exposed stigma may substantially decrease the costs associated with producing tomato hybrid seeds, thereby increasing their commercial utility.

## Data Availability

Data sharing not applicable to this article as no datasets were generated or analysed during the current study.
